# Validation of the Diagnosis Procedure Combination Database in Japan for ICU Research: A Multicenter Comparison With the Japanese Intensive care PAtient Database (JIPAD)

**DOI:** 10.2188/jea.JE20250425

**Published:** 2026-09-05

**Authors:** Hiroyuki Ohbe, Junji Kumasawa, Hiroshi Okamoto, Eiji Hashiba, Shinichiro Ohshimo, Takashi Tagami, Taisuke Yasaka, Satoru Hashimoto, Naoya Kobayashi, Daisuke Kudo

**Affiliations:** 1 Department of Emergency and Critical Care Medicine, Tohoku University Hospital, Sendai, Japan; 2 Department of Clinical Epidemiology and Health Economics, School of Public Health, The University of Tokyo, Tokyo, Japan; 3 Department of Critical Care Medicine, Sakai City Medical Center, Osaka, Japan; 4 Department of Critical Care Medicine, St. Luke’s International Hospital, Tokyo, Japan; 5 Division of Intensive Care, Hirosaki University Hospital, Aomori, Japan; 6 Department of Emergency and Critical Care Medicine, Graduate School of Biomedical and Health Sciences, Hiroshima University, Hiroshima, Japan; 7 Department of Emergency and Disaster Medicine, The Jikei University School of Medicine, Tokyo, Japan; 8 Department of Nursing Data Science, Graduate School of Medicine, The University of Tokyo, Tokyo, Japan; 9 ICU Collaboration Network (ICON), Tokyo, Japan; 10 Department of Anesthesiology and Perioperative Medicine, Tohoku University Graduate School of Medicine, Sendai, Japan; 11 Division of Emergency and Critical Care Medicine, Tohoku University Graduate School of Medicine, Sendai, Japan

**Keywords:** validation, administrative data, intensive care unit, Japan, registry

## Abstract

**Background:**

The Diagnosis Procedure Combination (DPC) database is Japan’s most widely used administrative inpatient dataset supporting epidemiological and health services research. While its validity is established for various diagnoses and procedures, accuracy for intensive care unit (ICU) variables has not been directly evaluated using a clinical registry as the gold standard.

**Methods:**

We conducted a multicenter retrospective validation study using four Japanese ICUs. Patient records from the national ICU registry, the Japanese Intensive care PAtient Database (JIPAD), were matched with corresponding DPC data, retaining only successfully matched patients to evaluate coding accuracy rather than case ascertainment. We assessed binary variables (demographics, comorbidities, diagnoses, interventions, ICU admission, mortality) and continuous variables (demographics, Sequential Organ Failure Assessment [SOFA] scores). We calculated sensitivity and specificity for binary variables and intraclass correlation coefficients (ICCs) for continuous variables.

**Results:**

We included 14,070 ICU admissions. Most binary variables, including demographics, major diagnostic categories, ICU interventions, and mortality, had high sensitivity and specificity (≥80%). Comorbidity specificity exceeded 95%, but sensitivity was <30% for several diseases. Sensitivity was 83.3% for invasive mechanical ventilation but low for noninvasive positive pressure ventilation (5.5%) and high-flow nasal cannula (36.1%), although specificity was high across respiratory supports (97.3–99.9%). SOFA scores showed moderate agreement (ICC, 0.61). Sensitivity/specificity were 99.1%/100.0% for in-hospital mortality and 96.2%/99.9% for ICU mortality.

**Conclusion:**

The DPC administrative inpatient database accurately captures most key ICU variables, but comorbidities, noninvasive respiratory support, and SOFA scores require caution. These findings support its use for ICU clinical and epidemiological research in Japan.

## INTRODUCTION

The Diagnosis Procedure Combination (DPC) data are administrative inpatient database in Japan, originally established primarily for hospital reimbursement and health system management.^1^^,^^2^ Its structured data on diagnoses, procedures, and outcomes have subsequently enabled numerous studies in clinical epidemiology, health services research, and health policy, including a growing body of research focused on intensive care units (ICUs).^3^^,^^4^ Among the various real-world data in Japan,^5^ the DPC database is currently the most extensively used for research purposes and exerts substantial influence on healthcare practice and policy; therefore, rigorous validation of its data is essential.

Since 2010, more than 36 validation studies of Japanese administrative healthcare data have evaluated the accuracy of DPC-coded diagnoses, procedures, and outcomes.^6^ The specificity of Charlson diseases in the DPC data exceeds 96%, while the sensitivity is below 50% for several diseases, whereas major procedures are recorded with both high sensitivity (>90%) and specificity (>90%).^7^ Disease-specific validation studies have confirmed these trends across stroke,^8^ cancer,^9^ diabetes,^10^ and cardiovascular disease,^11^^,^^12^ generally showing high positive predictive value (PPV) and specificity, with variable sensitivity depending on coding practices and disease definitions.

However, to the best of our knowledge, no validation studies have directly assessed the accuracy of Japanese administrative healthcare data, specifically for ICU-related variables. International validation studies have demonstrated that administrative data can reliably identify ICU interventions and hospital mortality; however, the accuracy of recording ICU interventions varies by country and coding system. In Canada, Garland et al found that hospital claims data could identify invasive mechanical ventilation (IMV) with excellent sensitivity, specificity, PPV, and negative predictive value (NPV)—all above 90%—when compared with a clinical registry, although vasoactive drug use was not accurately captured.^13^^,^^14^ In contrast, a United States study by Wunsch et al showed that, while administrative data reliably identified ICU admission and mortality, the sensitivity for detecting IMV was only 58%, despite high specificity (96%), leading to an underestimation of ventilated patients, particularly those with brief or perioperative ventilation.^15^ These results highlight that while ICU admission and mortality are generally robust in administrative data, the detection of specific interventions, such as IMV or vasoactive drugs, remains challenging and requires local validation for each healthcare system.

To address these gaps, the present study aimed to systematically validate key ICU-related variables in the DPC database using the national ICU registry (Japanese Intensive care PAtient Database [JIPAD]) in Japan as the gold standard. We specifically focused on evaluating the coding accuracy of ICU-related variables among patients whose ICU admission was confirmed using JIPAD.

## METHODS

### Study design and setting

This retrospective observational validation study used data from four intensive care units (ICUs) in Japan. The study is part of the JIPAD–DPC project, one of the official research activities conducted by the JIPAD Working Group of the Japanese Society of Intensive Care Medicine. Data were obtained from the JIPAD-DPC project, which links the JIPAD^16^ with the DPC database.^1^^,^^2^ In the JIPAD–DPC database, ICU identifiers are anonymized and cannot be used to identify individual ICU. This study followed the Strengthening the Reporting of Observational Studies in Epidemiology statement.^17^

### Data sources

Data were first collected retrospectively from JIPAD and then matched with the corresponding records from the DPC database. The JIPAD is a prospective, national ICU registry established by the Japanese Society of Intensive Care Medicine.^16^ All ICU admissions at the participating hospitals are mandatorily registered in JIPAD under standardized data entry protocols, and no ICU admissions are selectively excluded. To maintain the validity and reliability of the data, each ICU undergoes data verification upon joining JIPAD, followed by ongoing quality monitoring conducted by the JIPAD Working Group.^16^

The DPC database used in this study comprises administrative data collected primarily for billing purposes, including discharge abstracts and administrative claims data held individually by each participating hospital.^1^^,^^2^ The DPC payment system, developed by the Ministry of Health, Labour and Welfare (MHLW) of Japan, classifies patients into diagnostic groups for payment purposes. The DPC database contains information on patient demographics, clinical variables, admission and discharge details, diagnoses, surgeries and procedures, medications, and special reimbursements for specific conditions. Diagnoses were entered by the attending physicians according to the International Classification of Diseases, Tenth Revision (ICD-10). Suspected diagnoses can also be recorded but are marked as provisional. The DPC system categorizes diagnoses into six groups with a fixed number of entries: one each for “main diagnosis,” “admission-precipitating diagnosis,” “most resource-consuming diagnosis,” and “second most resource-consuming diagnosis,” and up to 10 each for “comorbidities present at time of admission” and “conditions arising after admission.” All services performed during hospitalization were recorded on a per-day basis according to the Japanese national fee schedule for reimbursement, with each service assigned a specific code. This allows for the detailed tracking of the types and timing of medical services throughout the hospitalization period.

### Study population

We included patients recorded in the JIPAD who were admitted to the ICU and subsequently discharged from the hospital. Patients from two ICUs were included between April 1, 2018, and May 1, 2024, while two other ICUs were included between April 1, 2020, and May 1, 2024, reflecting differences in the timing of their participation in the JIPAD. For these patients, the corresponding DPC data were linked within each ICU using dedicated software that matched records based on the date of birth, hospital admission date, and hospital discharge date, allowing a ±1 day discrepancy for hospital admission and discharge dates. ICU admission was not used as a linkage key. Because our aim was to evaluate coding accuracy rather than case ascertainment, DPC data were joined using an INNER JOIN strategy (ie, only records with matching information in both databases were retained), and unmatched JIPAD records were excluded. The resulting anonymized matched dataset was then collected and aggregated into the unified JIPAD-DPC database.

Patients with multiple ICU stays within the same hospitalization were excluded because JIPAD records each ICU stay as a separate entry, whereas DPC data are organized per hospitalization. Consequently, when a patient had multiple ICU admissions during one hospitalization, multiple DPC records could potentially be linked, making it difficult to determine which DPC record corresponds to which ICU stay. Additionally, admissions involving patients aged under 15 years were excluded because JIPAD stores pediatric cases separately, with distinct disease categories and severity scoring systems.

### Variables

Validation was conducted on two types of variables: binary and continuous variables. The binary variables included sex, postoperative status, emergency admission, comorbidities, major diagnostic categories, ICU interventions (including IMV), ICU admission, and mortality (both in-hospital and ICU). Continuous variables included age, height, weight, body mass index (BMI), Sequential Organ Failure Assessment (SOFA) score (both total and individual organ system components), length of IMV, and ICU length of stay (LOS). The definitions for each variable in the JIPAD database are available on the JIPAD homepage (Japanese only)^18^ (https://www.jipad.org/). The definitions used in the DPC database are summarized in Table 1.

**Table 1.** DPC definitions for binary and continuous variables

Variable	DPC File	DPC Definition and Extraction Logic
**Binary variables**		
Male sex	Format 1	Sex = ‘1’
Post-operative	EF file	L008 Closed-circuit general anesthesia using a mask or endotracheal intubation performed on the day of ICU admission or earlier.
Emergency ICU admission	Format 1	Admission is considered emergency if the admission code is ‘200’ or starts with ‘3’ (ie, ‘3**’).
Comorbidities		For each comorbidity, patients were defined as positive if any of the ten diagnosis fields for “comorbidities present at the time of admission” included an ICD-10 code as listed below:
AIDS	Format 1	B20–B24
Congestive heart failure	Format 1	I099, I110, I130, I132, I50
Respiratory failure	Format 1	J40–J47, J60–J70, I27
Hepatic failure	Format 1	K704, K711, K72
Cirrhosis	Format 1	B181, B182, K703, K717, K743–K746, K761, K766
Leukemia/multiple myeloma	Format 1	C90–C95
Lymphoma	Format 1	C81–C86, C96
Metastatic cancer	Format 1	C77–C80
Immunosuppression	Format 1	B24, C81, C84, C85, C88, C90–C96, D80–D89, Z94
Maintenance dialysis	Format 1	N185
Major diagnostic category		For each major diagnostic category, patients were defined as positive if any of the four diagnosis fields for “main diagnosis,” “admission-precipitating diagnosis,” “most resource-consuming diagnosis,” and “second most resource-consuming diagnosis” included an ICD-10 code as listed below:
Cardiovascular	Format 1	I00–I52, I70-I99, R570
Respiratory	Format 1	C30–C39, J00–J99, R092
Gastrointestinal	Format 1	C15–C21, K00–K93
Neurological	Format 1	G00–G99, I60–I69, C70–C72
Sepsis	Format 1	Positive if the SOFA score for sepsis-related variables was not missing.
Trauma	Format 1	S00–T98, T00, T01
Metabolic	Format 1	E00–E90, T36–T39, T40–T65
Hematological	Format 1	C81–C96, D50–D89
Genitourinary	Format 1	N00–N99, C60–C68
Musculoskeletal	Format 1	M00–M99, C40–C41
Gynecological	Format 1	O00–O99, C51–C58
ICU treatment		For each ICU treatment, the variable was considered positive if the corresponding procedure’s Kubun code was billed on any day between the date of ICU admission and the date of ICU discharge.
Arterial catheterization	EF file	D225 Invasive arterial blood pressure monitoring
HFNC	EF file	J026-4 High-flow therapy
NPPV	EF file	J026 Intermittent positive pressure ventilation, J026-2 Nasal mask ventilation, J045 Artificial respiration for nasal mask ventilation (corresponding billing codes are “140010050”, “140024250”, “140039550”, “140039650”, “140064750”)
Tracheostomy	EF file	K386 Tracheostomy
IABP	EF file	K600 Intra-aortic balloon pumping (IABP)
PCPS (VA or VV ECMO)	EF file	K602 Percutaneous cardiopulmonary support
Intermittent RRT	EF file	J038 Renal replacement therapy
Continuous RRT	EF file	J038-2 Continuous renal replacement therapy
Plasma exchange	EF file	J039 Plasma exchange
PMX	EF file	J041 Adsorptive blood purification therapy
Catecholamines	EF file	For each catecholamines, the variable was considered positive if the corresponding drug code was billed on any day between the date of ICU admission and the date of ICU discharge.
Dopamine	EF file	Dopamine
Dobutamine	EF file	Dobutamine
Noradrenaline	EF file	Noradrenaline
Adrenaline	EF file	Adrenaline
IMV in the ICU		
DPC (J045)	EF file	All codes for J045 Artificial respiration
DPC (J045 w/o 30 min)	EF file	All codes for J045 Artificial respiration, excluding codes corresponding to artificial respiration for up to 30 minutes (corresponding billing codes are “140009310”, “140009550”, “140009750”, “140009950”, “140010150”).
DPC (J045 or J044)	EF file	All codes for J045 Artificial respiration and J044 Endotracheal intubation for lifesaving
DPC (J045 or L008)	EF file	All codes for J045 Artificial respiration and L008 Closed-circuit general anesthesia using a mask or endotracheal intubation
ICU admission		
DPC (Format 1)	Format 1	Positive if the SOFA score for ICU-related variables were not missing.
DPC (billing)	EF file	Positive if A301 ICU management fee was billed on any day.
DPC (ward-code)	EF file	Positive if any billing was recorded in the EF file for a ward that is eligible to bill the A301 ICU management fee.
DPC (H-file)	H file	Positive if a record for ASS0040 “Evaluation form for severity and medical/nursing needs in specialized intensive care” was present in the nursing acuity data stored in the H-file.
In-hospital mortality	Format 1	Positive if the discharge status was coded as 6 (“death due to the disease that consumed the most medical resources”) or 7 (“death due to causes other than the disease that consumed the most medical resources”).
ICU mortality	Format 1, EF file	Positive if the discharge status was coded as 6 (“death due to the disease that consumed the most medical resources”) or 7 (“death due to causes other than the disease that consumed the most medical resources”), and if the date of discharge was the same as the last day the A301 ICU management fee was billed.
**Continuous variables**		
Age, years	Format 1	Age (years) was calculated using the date of birth and the date of hospital admission.
Height, cm	Format 1	Height at admission (in centimeters, rounded).
Weight, kg	Format 1	Weight at admission (in kilograms, to one decimal place, rounded).
BMI	Format 1	BMI was calculated from height and weight at admission.
Total SOFA score	Format 1	Recorded as the sum of six organ subscores on ICU admission, using the highest daily value (or the earliest if tied).
Each organ system		Each subscore (respiration, coagulation, liver, cardiovascular, CNS, renal) was recorded as a continuous variable, using the highest daily value.
Respiration	Format 1	Respiration
Coagulation	Format 1	Coagulation
Liver	Format 1	Liver
Cardiovascular	Format 1	Cardiovascular
Central Nervous	Format 1	Central Nervous
Renal	Format 1	Renal
Length of IMV		
DPC (billing)	EF file	Length of IMV was calculated as the total number of days on which any billing code for J045 Artificial respiration was recorded in the EF file during periods when the A301 ICU management fee was billed.
Length of ICU stay		
DPC (Format 1)	Format 1	Calculated as the number of days between ICU admission and ICU discharge dates recorded in Format 1.
DPC (billing)	EF file	Calculated as the total number of days on which the A301 ICU management fee was billed in the EF file.
DPC (ward-code)	EF file	Calculated as the total number of days with any billing record in the EF file for a ward eligible to bill the A301 ICU management fee.
DPC (H-file)	H file	Calculated as the total number of days with records of ASS0040 “Evaluation form for severity and medical/nursing needs in specialized intensive care” in the nursing acuity data stored in the H-file.

BMI, body mass index; CNS, central nervous system; DPC, Diagnosis Procedure Combination; ECMO, extracorporeal membrane oxygenation; EF file, Electronic Fee file; H-file, nursing acuity file; HFNC, high-flow nasal cannula; IABP, intra-aortic balloon pumping; ICU, intensive care unit; IMV, invasive mechanical ventilation; NPPV, noninvasive positive pressure ventilation; PCPS, percutaneous cardiopulmonary support; PMX, polymyxin B hemoperfusion; RRT, renal replacement therapy; SOFA, Sequential Organ Failure Assessment; VA, veno-arterial; VV, veno-venous.

When analyzing IMV in the ICU and ICU admission, multiple identification algorithms were applied to the DPC database. Specifically, IMV in the ICU was identified in the DPC database using four different claims-based definitions: (1) J045 “artificial ventilation” procedure code alone, (2) J045 code with a ≥30-minute time restriction, (3) either the J045 or J044 “lifesaving endotracheal intubation” codes, and (4) either the J045 or L008 “general anesthesia” codes. ICU admission in the DPC database was identified using four different definitions: (1) billing codes for ICU reimbursement, (2) ward codes indicating ICU, (3) detailed ICU status in the discharge summary (Format 1), and (4) the H-file containing nursing acuity data.^1^^,^^2^ These alternative approaches were evaluated for accuracy against the JIPAD gold standard. The length of IMV was validated using (1) the J045 procedure code alone in the DPC database.

### Statistical analysis

For binary variables, sensitivity, specificity, PPV, NPV, and Cohen’s kappa were calculated. For ICU admission, we calculated concordance proportions, because sensitivity and specificity could not be calculated. For continuous variables, mean differences, exact match percentages, match percentages within ±1 unit, and intraclass correlation coefficients (ICC) were calculated. Cohen’s kappa is a statistic that measures the agreement between two raters or data sources, accounting for agreement occurring by chance; values above 0.8 are generally considered to indicate almost perfect agreement.^19^ The ICC is a reliability index that quantifies the degree of agreement or consistency between two or more measurements; values above 0.75 are generally interpreted as indicating good reliability.^20^ All analyses were performed using the Stata/MP 17.0 software (StataCorp, College Station, TX, USA).

### Missing data

No missing values were found for the binary variables. For the calculation of mean differences and ICC, only cases with non-missing values in both JIPAD and DPC were included in the analysis. For exact match and match-within-one-unit analyses, cases with missing values in JIPAD were excluded from the analysis, whereas cases with missing values in DPC were classified as non-matched.

### Ethics approval and consent to participate

The Research Ethics Committee of Hiroshima University approved this study (approval number: E2023-0279; approval date: April 9, 2024). The requirement for written informed consent was waived because all data were anonymized.

## RESULTS

**Figure 1.**
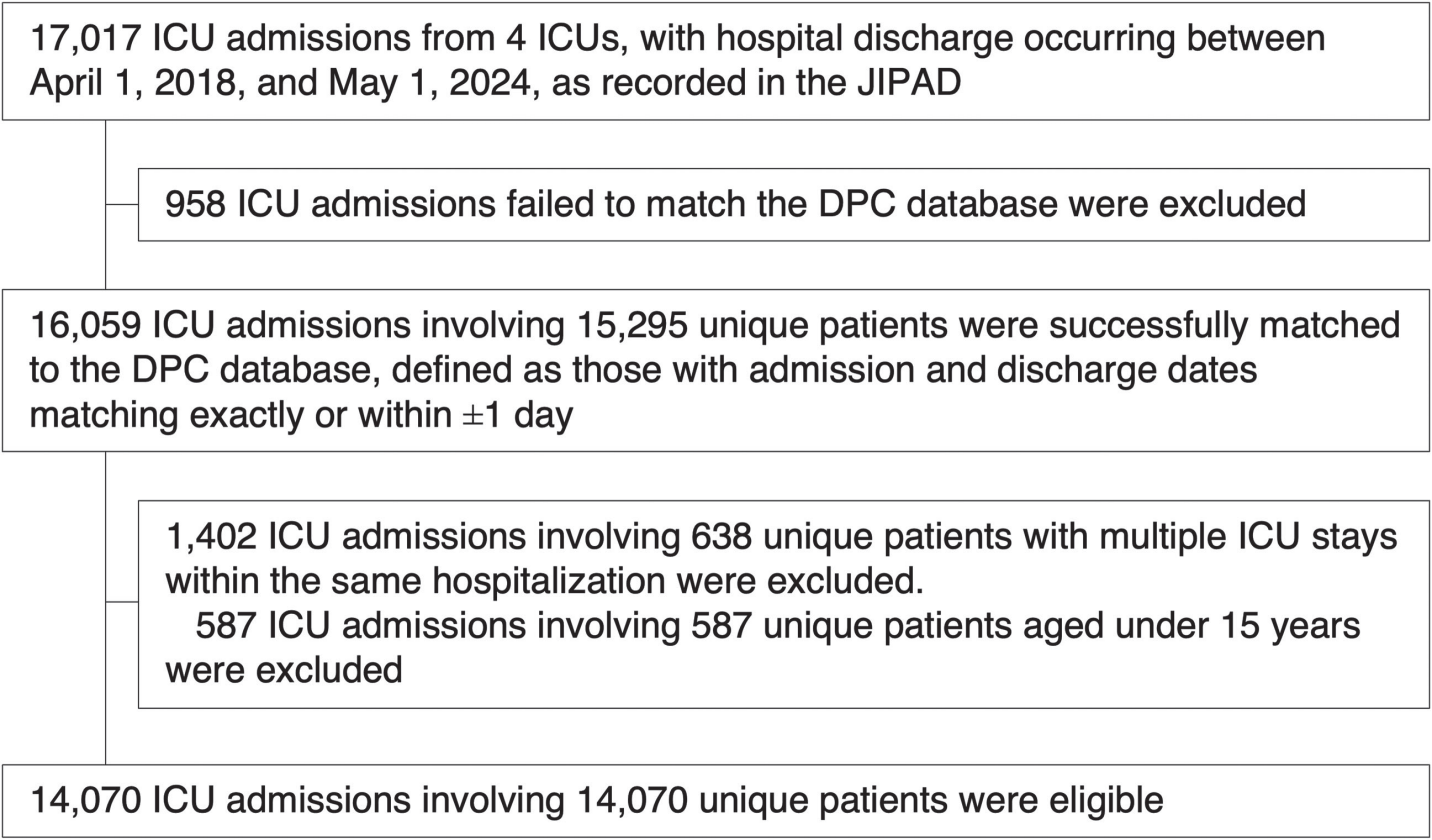
Patient flow chart. DPC, Diagnosis Procedure Combination; ICU, intensive care unit; JIPAD, Japanese Intensive care PAtient Database.

Among 17,017 ICU admissions from four ICUs recorded in the JIPAD, 958 (5.6%) failed to match the DPC database using dedicated software matching based on the date of birth, hospital admission date, and hospital discharge date, primarily due to >1-day discrepancies in hospital admission or discharge dates. After excluding patients with multiple ICU stays within the same hospitalization (*n* = 1,402) and those aged under 15 years (*n* = 587), the final cohort comprised 14,070 ICU admissions (Figure 1). Of these, 54% were contributed by ICU A, followed by 21% from ICU B, 13% from ICU C, and 12% from ICU D.

In the gold standard cohort from JIPAD, the mean age was 65.5 years (standard deviation [SD], 14.9), and 8,219 patients (58.4%) were male (Table 2 and Table 3). The majority of admissions were postoperative (78.7%), and 31.0% were emergency ICU admissions. The most common major diagnostic category was cardiovascular (23.7%), followed by gastrointestinal (20.9%) and respiratory (18.8%) diseases. The mean total SOFA score within the first 24 hours of ICU admission was 4.4 (SD, 3.4). Regarding ICU interventions, 98.5% of patients underwent arterial catheterization, 29.2% received IMV, 2.4% received noninvasive positive pressure ventilation (NPPV), and 7.6% received high-flow nasal cannula (HFNC). The in-hospital and ICU mortality were 4.6% and 2.0%, respectively. The mean length of IMV and ICU stay were 4.6 days and 3.1 days, respectively, calculated as the difference between the YMDHS (year-month-day-hour-second) end and start times, and then rounded up.

**Table 2.** Validation for binary variables^a^

	JIPAD		DPC		Sensitivity, (%)	Specificity, (%)	PPV, (%)	NPV, (%)	Cohen’s kappa
	
	Missing	*N* = 14,070	Missing	*N* = 14,070					
Male sex	0	8,219 (58.4%)	0	8,215 (58.4%)	100.0	100.0	100.0	99.9	1.00
Post-operative	0	11,077 (78.7%)	0	10,781 (76.6%)	94.6	89.9	97.2	81.8	0.82
Emergency ICU admission	0	4,359 (31.0%)	0	4,569 (32.5%)	86.2	91.6	82.2	93.7	0.77
Comorbidities									
AIDS	0	1 (0.0%)	0	5 (0.0%)	100.0	100.0	20.0	100.0	0.33
Congestive heart failure	0	151 (1.1%)	0	1,254 (8.9%)	45.0	91.5	5.4	99.4	0.08
Respiratory failure	0	194 (1.4%)	0	846 (6.0%)	30.4	94.3	7.0	99.0	0.09
Hepatic failure	0	73 (0.5%)	0	27 (0.2%)	11.0	99.9	29.6	99.5	0.16
Cirrhosis	0	129 (0.9%)	0	307 (2.2%)	31.0	98.1	13.0	99.4	0.17
Leukemia/multiple myeloma	0	80 (0.6%)	0	36 (0.3%)	22.5	99.9	50.0	99.6	0.31
Lymphoma	0	84 (0.6%)	0	39 (0.3%)	20.2	99.8	43.6	99.5	0.27
Metastatic cancer	0	553 (3.9%)	0	320 (2.3%)	17.4	98.3	30.0	96.7	0.20
Immunosuppression	0	1,011 (7.2%)	0	136 (1.0%)	3.1	99.2	22.8	93.0	0.04
Maintenance dialysis	0	547 (3.9%)	0	197 (1.4%)	28.5	99.7	79.2	97.2	0.41
Major diagnostic category									
Cardiovascular	0	3,332 (23.7%)	0	3,277 (23.3%)	89.2	97.2	90.7	96.7	0.87
Respiratory	0	2,648 (18.8%)	0	1,649 (11.7%)	53.5	98.0	86.0	90.1	0.60
Gastrointestinal	0	2,936 (20.9%)	0	2,811 (20.0%)	87.3	97.8	91.1	96.7	0.86
Neurological	0	1,479 (10.5%)	0	1,081 (7.7%)	50.8	97.4	69.6	94.4	0.55
Sepsis	0	264 (1.9%)	0	720 (5.1%)	74.2	96.2	27.2	99.5	0.38
Trauma	0	43 (0.3%)	0	388 (2.8%)	51.2	97.4	5.7	99.8	0.10
Metabolic	0	458 (3.3%)	0	492 (3.5%)	45.0	97.9	41.9	98.1	0.41
Hematological	0	72 (0.5%)	0	305 (2.2%)	75.0	98.2	17.7	99.9	0.28
Genitourinary	0	888 (6.3%)	0	1,283 (9.1%)	83.1	95.9	57.5	98.8	0.65
Musculoskeletal	0	1,245 (8.8%)	0	923 (6.6%)	49.6	97.6	67.0	95.2	0.54
Gynecological	0	680 (4.8%)	0	569 (4.0%)	66.3	99.1	79.3	98.3	0.71
ICU treatment									
Arterial catheterization	0	13,861 (98.5%)	0	13,448 (95.6%)	96.7	81.3	99.7	27.3	0.40
NPPV	0	344 (2.4%)	0	34 (0.2%)	5.5	99.9	55.9	97.7	0.10
HFNC	0	1,068 (7.6%)	0	471 (3.3%)	36.1	99.3	82.0	95.0	0.48
Tracheostomy	0	272 (1.9%)	0	537 (3.8%)	92.1	99.1	77.8	99.7	0.84
IABP	0	231 (1.6%)	0	238 (1.7%)	93.1	99.8	90.3	99.9	0.92
PCPS (VA or VV ECMO)	0	184 (1.3%)	0	155 (1.1%)	76.6	99.9	91.0	99.7	0.83
Intermittent RRT	0	502 (3.6%)	0	687 (4.9%)	86.1	98.1	62.9	99.5	0.71
Continuous RRT	0	695 (4.9%)	0	715 (5.1%)	94.8	99.6	92.2	99.7	0.93
Plasma exchange	0	52 (0.4%)	0	59 (0.4%)	78.8	99.9	69.5	99.9	0.74
PMX	0	31 (0.2%)	0	35 (0.2%)	54.8	99.9	48.6	99.9	0.51
Catecholamines									
Dopamine	0	119 (0.8%)	0	160 (1.1%)	68.9	99.4	51.3	99.7	0.58
Dobutamine	0	1,427 (10.1%)	0	1,586 (11.3%)	72.3	95.6	65.1	96.8	0.65
Noradrenaline	0	2,983 (21.2%)	0	4,707 (33.5%)	97.5	83.8	61.8	99.2	0.67
Adrenaline	0	98 (0.7%)	0	2,655 (18.9%)	92.9	81.6	3.4	99.9	0.05
IMV in the ICU									
vs DPC (J045)	0	4,108 (29.2%)	0	3,694 (26.3%)	83.3	97.3	92.7	93.4	0.83
vs DPC (J045 w/o 30 min)	0	4,108 (29.2%)	0	3,559 (25.3%)	80.9	97.7	93.4	92.6	0.82
vs DPC (J045 or J044)	0	4,108 (29.2%)	0	3,719 (26.4%)	83.9	97.3	92.7	93.6	0.84
vs DPC (J045 or L008)	0	4,108 (29.2%)	0	12,367 (87.9%)	97.5	16.1	32.4	94.1	0.09
In-hospital mortality	0	654 (4.6%)	0	652 (4.6%)	99.1	100.0	99.4	100.0	0.99
ICU mortality	0	287 (2.0%)	0	289 (2.1%)	96.2	99.9	95.5	99.9	0.96

AIDS, acquired immunodeficiency syndrome; DPC, Diagnosis Procedure Combination; ECMO, extracorporeal membrane oxygenation; HFNC, high-flow nasal cannula; IABP, intra-aortic balloon pumping; ICU, intensive care unit; IMV, invasive mechanical ventilation; JIPAD, Japanese Intensive care Patient Database; NPV, negative predictive value; NPPV, noninvasive positive pressure ventilation; PCPS, percutaneous cardiopulmonary support; PMX, polymyxin B hemoperfusion; PPV, positive predictive value; RRT, renal replacement therapy; VA, veno-arterial; VV, veno-venous.

^a^All validity metrics in this table were calculated only among successfully linked JIPAD–DPC pairs (INNER JOIN). These values represent coding accuracy conditional on successful linkage, not overall case-ascertainment performance of the DPC database.

**Table 3.** Validation for continuous variables

	JIPAD		DPC		Mean difference	Exact match	Match within 1	ICC
	
	Missing	*N* = 14,070	Missing	*N* = 14,070				
Age, years	0	65.5 (14.9)	1	65.4 (14.9)	0.01	0.99	1.00	1.00
Height, cm	21	160.9 (9.7)	0	160.0 (16.1)	0.80	0.69	0.96	0.59
Weight, kg	6	61.0 (14.3)	0	61.1 (14.7)	−0.11	0.43	0.64	0.96
BMI, kg/m^2^	21	23.5 (5.4)	92	23.5 (4.6)	0.00	0.56	0.85	0.80
Total SOFA score	0	4.4 (3.4)	0	3.0 (3.5)	1.46	0.22	0.54	0.61
Each organ system								
Respiration	0	0.9 (1.0)	0	0.9 (1.1)	−0.04	0.63	0.85	0.54
Coagulation	0	0.7 (0.9)	0	0.5 (0.8)	0.18	0.85	0.96	0.73
Liver	0	0.4 (0.7)	0	0.3 (0.6)	0.14	0.81	0.97	0.65
Cardiovascular	0	1.2 (1.3)	0	0.6 (1.2)	0.65	0.45	0.88	0.55
Central Nervous	0	0.3 (0.9)	0	0.4 (0.9)	−0.06	0.78	0.91	0.42
Renal	0	0.6 (1.2)	0	0.3 (0.9)	0.26	0.84	0.92	0.61
Length of IMV, days^a^								
vs DPC (J045)	9,963	4.6 (10.5)	10,639	3.9 (4.7)	1.20	0.44	0.65	0.55
Length of ICU stay, days^a^								
vs DPC (Format 1)	0	3.1 (6.9)	2,518	3.7 (6.8)	−0.89	0.09	0.81	0.89
vs DPC (billing)	0	3.1 (6.9)	604	2.6 (3.8)	0.24	0.81	0.92	0.62
vs DPC (ward-code)	0	3.1 (6.9)	420	3.0 (7.4)	0.01	0.83	0.95	0.92
vs DPC (H-file)	0	3.1 (6.9)	4,650	3.7 (8.6)	−0.26	0.55	0.63	0.91

BMI, body mass index; DPC, Diagnosis Procedure Combination; ICC, intraclass correlation coefficient; ICU, intensive care unit; IMV, invasive mechanical ventilation; JIPAD, Japanese Intensive care Patient Database; SOFA, Sequential Organ Failure Assessment.

^a^Length of IMV and ICU stay in the JIPAD were calculated as the difference between the YMDHS (year-month-day-hour-second) end and start times and then rounded up to the nearest whole day.

The results of the validation of binary variables of DPC data against the JIPAD gold standard cohort are shown in Table 2. Male sex was recorded with high sensitivity and specificity (100.0% and 100.0%, respectively). Postoperative status was also accurately captured (sensitivity, 94.6%; specificity, 89.9%), while emergency ICU admission showed somewhat lower but still acceptable validity (sensitivity, 86.2%; specificity, 91.6%). Most comorbidities showed high specificity (generally >95%) but low sensitivity (generally >30%). All major diagnostic categories exhibited very high specificity (generally >97%), while sensitivity was high for cardiovascular and gastrointestinal diseases (87–89%) and moderate for other categories, ranging from 45.0% to 83.1%. Most major ICU interventions demonstrated high sensitivity and specificity. However, the sensitivity was lower for NPPV (5.5%) and HFNC (36.1%), although the specificity for these interventions remained high. For IMV, the sensitivity and specificity were 83.3% and 97.3%, respectively, when using the J045 “artificial ventilation” code. Restricting the J045 code to cases lasting ≥30 minutes increased specificity by only 0.4% but reduced sensitivity by 2.4%. Adding the J044 “lifesaving endotracheal intubation” code raised the sensitivity by only 0.6%. Adding the L008 “general anesthesia” code increased the sensitivity but markedly reduced the specificity. In-hospital and ICU mortality were both detected with high accuracy (in-hospital: sensitivity, 99.1%; specificity 100.0%; ICU: sensitivity, 96.2%; specificity 99.9%).

For ICU admissions recorded in JIPAD, billing codes and ward codes in the DPC database showed high concordance (95.7% and 97.0%, respectively). In contrast, concordance was 82.1% using the discharge summary from the Format 1 and 67.0% using the nursing acuity data from the H-file.

The results of the validation of the continuous variables are shown in Table 3. For age, height, weight, and BMI, the agreement between JIPAD and DPC was strong, with mean differences close to zero, high exact match rates, and excellent ICC. For the total SOFA score, the mean difference was 1.46, with a lower ICC of 0.61. Because of the large proportion of missing data for the length of IMV and ICU stay in the DPC database, the match rate may provide a more robust indicator of validity than the mean difference or ICC. For the length of IMV, the mean difference was 1.20 days, with an exact match percentage of 44% and a match percentage within ±1 day of 65%. For ICU LOS, the match rate within ±1 day between JIPAD and DPC (ward-code) was the highest among the four DPC-based definitions, at 95%, with a mean difference of only 0.01 day, indicating good agreement. In comparison, the match rates were 81%, 92%, and 63% for DPC (Format 1), DPC (billing), and DPC (H-file), respectively.

## DISCUSSION

This multicenter validation study demonstrated that the administrative DPC database captures a wide range of ICU clinical variables with high accuracy compared with the JIPAD gold standard registry. Most binary outcomes, including patient demographics, major diagnostic categories, ICU interventions, and mortality, showed excellent sensitivity and specificity. Continuous variables, such as age and height demonstrated nearly perfect agreement, and ICU LOS, also exhibited high concordance. These findings suggest that the DPC can serve as a reliable resource for ICU research, although some caution is warranted for variables with known differences in definitions or recording practices.

The validity of the comorbidity data was notably limited by low sensitivity, despite specificity exceeding 95% in most cases. This finding suggests that while the presence of comorbidities recorded in the DPC is reliable, many comorbidities are likely underreported when compared with clinical registry data. This finding is consistent with the results of a previous work by Yamana et al, who reported similarly low sensitivity for DPC-coded comorbidities in a nationwide validation study.^7^ This limitation may be due to the administrative nature of the DPC, where the primary purpose of coding is billing rather than clinical details. Consequently, comorbidities that do not directly influence reimbursement or LOS may be omitted. In addition, we note that JIPAD collects pre-existing conditions based on its own definitions, which are not aligned with ICD-10 classifications. Therefore, some discrepancies with DPC coding are inherent and unavoidable. As such, reliance on comorbidity variables from DPC data for risk adjustment, case-mix classification, or epidemiologic modeling may lead to substantial misclassification and underestimation of patient severity.

For ICU interventions, most procedures were captured with high sensitivity and specificity; however, NPPV and HFNC were the exceptions. The sensitivity for NPPV and HFNC was only 5.5% and 36.1%, respectively, despite specificity remaining high. This discrepancy is likely due to the DPC reimbursement rules, which do not allow the simultaneous billing of IMV and NPPV or HFNC on the same day. Consequently, when both IMV and NPPV or HFNC are provided, only IMV tends to be recorded in the DPC, resulting in substantial underreporting of NPPV and HFNC use. In addition, under the Japanese reimbursement criteria, the use of NPPV can be billed as IMV in cases of acute respiratory failure with a ratio of arterial oxygen partial pressure to fractional inspired oxygen (PaO_2_/FiO_2_) ≤300 mm Hg or partial pressure of arterial carbon dioxide (PaCO_2_) ≥45 mm Hg, which further complicates the identification of NPPV. Furthermore, HFNC can only be billed in cases of acute respiratory failure with a PaO_2_ ≤60 mm Hg or peripheral oxygen saturation (SpO_2_) ≤90%. Consequently, analyses using DPC data alone are likely to significantly underestimate the actual use of NPPV or HFNC, which may bias studies on respiratory management, resource utilization, or outcomes related to these interventions.

For IMV using J045 “artificial ventilation” billing code, sensitivity and specificity were 83.3% and 97.3%, respectively, indicating good validity. These values are comparable to those reported in international validation studies of IMV using administrative data, which have typically found sensitivities ranging from 77% to 96% and specificities from 92% to 99%.^13^^,^^15^ This suggests that the accuracy of IMV coding in the DPC database is similar to that observed in large-scale datasets from other countries. While recent commentaries have raised concerns that the J045 “artificial ventilation” billing code may be too broad to accurately identify IMV—potentially including bag-valve-mask ventilation, noninvasive ventilation, or even CPR-related ventilation^21^^,^^22^—our study addressed these limitations by validating DPC claims data directly against the clinical gold standard of the JIPAD registry. We found that using the J045 code alone—without combining it with J044 “lifesaving endotracheal intubation,” L008 “general anesthesia,” or restricting J045 to cases lasting ≥30 minutes—was sufficient to achieve good validity for identifying IMV. Although the length of IMV may be slightly underestimated, our findings provide a robust foundation for future research utilizing DPC data in IMV-related studies.

With respect to the SOFA score, the mean difference between DPC and JIPAD data was large, and the ICC was moderate, reflecting only a fair agreement. This finding likely arises from the difference in the timing and method of SOFA score calculation between the databases: in JIPAD, SOFA is calculated as the worst value within the first 24 hours of ICU admission, whereas in the DPC, the score is recorded only on the day of ICU admission. This difference in timing and methodology may lead to a significant underestimation in DPC data, particularly in patients whose condition rapidly deteriorates after ICU admission. Given that the original SOFA definition by Vincent et al specifies the use of the worst value within 24 hours,^23^ the DPC-based SOFA should be interpreted with caution for outcome modeling or risk adjustment.

Finally, the calculation of the length of ICU stay in the DPC database varies by method. Our results show that using ward codes in the DPC database yielded the best agreement with JIPAD clinical data (95% match within ±1 day; mean difference 0.01 day). In contrast, other DPC-based methods, such as ICU status in discharge summary (Format 1) and nursing acuity data in the H-file, have high rates of missing data. Billing-based length of ICU stay is generally capped at 14 days, leading to a systematic underestimation for patients with longer ICU stays. Therefore, the ward code method is recommended for accurately estimating the length of ICU stay from DPC data.

ICU admission showed generally high concordance between the DPC and JIPAD databases, with ward codes demonstrating the highest at 97.0%. However, because this analysis reflects coding agreement rather than diagnostic validity, these concordance proportions should not be interpreted as true sensitivity, nor do they establish the performance of case ascertainment for ICU admission across the full DPC population. To our knowledge, no previous study has validated ICU admission coding in the DPC database. Some DPC items, such as Format 1 and H-file, may technically be recorded even for non-ICU patients. ICU billing codes are also subject to institutional differences and periodic revisions in the national fee schedule, and some patients who were actually admitted to an ICU may become ineligible for billing if they do not meet these administrative requirements. These factors should also be considered when interpreting our results.

There are several limitations to this study. First, our validation focused exclusively on ICU patients whose admission was confirmed in JIPAD. Accordingly, our findings suggest that DPC data can be cautiously used in research limited to ICU-admitted patients. Second, because unmatched DPC records were excluded to focus on coding accuracy, our estimates reflect agreement conditional on successful linkage and may introduce some bias if linkage errors correlate with coding errors. Third, the study was conducted in four tertiary care institutions actively participating in the JIPAD registry and maintaining high standards of data recording and coding practices. These institutions may not be representative of smaller, community-based, or JIPAD non-participating institutions. Institutional variations in coding practices, patient populations, and case-mix may influence the validity of DPC variables. Fourth, several clinically important variables, such as the distinction between venous-arterial and veno-venous extracorporeal membrane oxygenation, Impella use, transfusion, or functional outcomes, were not routinely captured in both databases and could not be evaluated. Finally, changes in DPC definitions, reimbursement policies, and data structures over time may affect the reproducibility of these findings in the future.

In conclusion, this multicenter validation study demonstrated that the Japanese DPC administrative inpatient database provides accurate information for most key ICU variables, including patient demographics, diagnostic categories, interventions, and mortality, compared with the JIPAD clinical registry. While DPC data are suitable for a wide range of intensive care research, some variables—especially comorbidities, noninvasive respiratory support, and SOFA scores—require careful interpretation.

## References

[1] Yasunaga H. Updated information on the Diagnosis Procedure Combination data. Ann Clin Epidemiol. 2024;6:106–110. 10.37737/ace.24015 39726797 PMC11668689

[2] Yasunaga H. Real World Data in Japan: Chapter II The Diagnosis Procedure Combination Database. Ann Clin Epidemiol. 2019;1:76–79. 10.37737/ace.1.3_76

[3] Ohbe H, Sasabuchi Y, Yamana H, Matsui H, Yasunaga H. Intensive care unit versus high-dependency care unit for mechanically ventilated patients with pneumonia: a nationwide comparative effectiveness study. Lancet Reg Health West Pac. 2021;13:100185. 10.1016/j.lanwpc.2021.100185 34527980 PMC8350066

[4] Ohbe H, Matsui H, Fushimi K, Yasunaga H. Epidemiology of chronic critical illness in Japan: a nationwide inpatient database study. Crit Care Med. 2021;49:70–78. 10.1097/CCM.0000000000004723 33177360

[5] Sato S, Yasunaga H. A review of studies using Japanese nationwide administrative claims databases. Ann Clin Epidemiol. 2023;5:58–64. 10.37737/ace.23008 38505730 PMC10944998

[6] Yamana H, Konishi T, Yasunaga H. Validation studies of Japanese administrative health care data: a scoping review. Pharmacoepidemiol Drug Saf. 2023;32:705–717. 10.1002/pds.5636 37146098

[7] Yamana H, Moriwaki M, Horiguchi H, Kodan M, Fushimi K, Yasunaga H. Validity of diagnoses, procedures, and laboratory data in Japanese administrative data. J Epidemiol. 2017;27:476–482. 10.1016/j.je.2016.09.009 28142051 PMC5602797

[8] Omama S, Tanno K, Inoue Y, . The potential of a stroke registry using diagnosis procedure combination data from all hospitals in a Japanese prefecture. Cerebrovasc Dis. 2022;51:447–452. 10.1159/000520668 35081532

[9] Shigemi D, Morishima T, Yamana H, Yasunaga H, Miyashiro I. Validity of initial cancer diagnoses in the Diagnosis Procedure Combination data in Japan. Cancer Epidemiol. 2021;74:102016. 10.1016/j.canep.2021.102016 34450452

[10] Kanehara R, Goto A, Goto M, . Validation study of diabetes definitions using Japanese Diagnosis Procedure Combination data among hospitalized patients. J Epidemiol. 2023;33:165–169. 10.2188/jea.JE20210024 34275972 PMC9939922

[11] Shima D, Ii Y, Higa S, . Validation of novel identification algorithms for major adverse cardiovascular events in a Japanese claims database. J Clin Hypertens (Greenwich). 2021;23:646–655. 10.1111/jch.14151 33369149 PMC8029538

[12] Nakai M, Iwanaga Y, Sumita Y, . Validation of acute myocardial infarction and heart failure diagnoses in hospitalized patients with the nationwide claim-based JROAD-DPC database. Circ Rep. 2021;3:131–136. 10.1253/circrep.CR-21-0004 33738345 PMC7956876

[13] Garland A, Marrie RA, Wunsch H, Yogendran M, Chateau D. Accuracy of administrative hospital data to identify use of life support modalities. A Canadian study. Ann Am Thorac Soc. 2020;17:229–235. 10.1513/AnnalsATS.201902-106OC 32003608

[14] Garland A, Yogendran M, Olafson K, Scales DC, McGowan KL, Fransoo R. The accuracy of administrative data for identifying the presence and timing of admission to intensive care units in a Canadian province. Med Care. 2012;50:e1–e6. 10.1097/MLR.0b013e318245a754 22270100

[15] Wunsch H, Kramer A, Gershengorn HB. Validation of intensive care and mechanical ventilation codes in medicare data. Crit Care Med. 2017;45:e711–e714. 10.1097/CCM.0000000000002316 28403118 PMC6557134

[16] Irie H, Okamoto H, Uchino S, . The Japanese Intensive care PAtient Database (JIPAD): a national intensive care unit registry in Japan. J Crit Care. 2020;55:86–94. 10.1016/j.jcrc.2019.09.004 31715536

[17] von Elm E, Altman DG, Egger M, . The Strengthening the Reporting of Observational Studies in Epidemiology (STROBE) statement: guidelines for reporting observational studies. Ann Intern Med. 2007;147:573–577. 10.7326/0003-4819-147-8-200710160-00010 17938396

[18] The Japanese Intensive care PAtient Database (JIPAD) https://www.jipad.org/. Accessed 26.07.2025.

[19] Cohen J. A coefficient of agreement for nominal scales. Educ Psychol Meas. 1960;20:37–46. 10.1177/001316446002000104

[20] Koo TK, Li MY. A guideline of selecting and reporting intraclass correlation coefficients for reliability research. J Chiropr Med. 2016;15:155–163. 10.1016/j.jcm.2016.02.012 27330520 PMC4913118

[21] Sakamoto A, Inoue Y. The method to identify invasive mechanical ventilation with Japanese claim data. J Intensive Care. 2024;12:48. 10.1186/s40560-024-00760-0 39529189 PMC11555860

[22] Ohbe H, Shime N, Yamana H, . Reply to the comment by Sakamoto et al. on “The method to identify invasive mechanical ventilation with Japanese claim data”. J Intensive Care. 2024;12:54. 10.1186/s40560-024-00767-7 39710727 PMC11665237

[23] Vincent JL, Moreno R, Takala J, . The SOFA (Sepsis-related Organ Failure Assessment) score to describe organ dysfunction/failure. On behalf of the Working Group on Sepsis-Related Problems of the European Society of Intensive Care Medicine. Intensive Care Med. 1996;22:707–710. 10.1007/BF01709751 8844239

